# Adamantane-Resistant Influenza A Viruses in the World (1902–2013): Frequency and Distribution of M2 Gene Mutations

**DOI:** 10.1371/journal.pone.0119115

**Published:** 2015-03-13

**Authors:** Guoying Dong, Chao Peng, Jing Luo, Chengmin Wang, Le Han, Bin Wu, Guangju Ji, Hongxuan He

**Affiliations:** 1 College of Global Change and Earth System Science, Beijing Normal University, Beijing, China; 2 National Research Center For Wildlife Born Diseases, Key Laboratory of Animal Ecology and Conservation Biology, Institute of Zoology, Chinese Academy of Sciences, Beijing, China; 3 National Laboratory of Biomacromolecules, Institute of Biophysics of Chinese Academy of Sciences, Beijing, China; University of Edinburgh, UNITED KINGDOM

## Abstract

Adamantanes (amantadine and rimantadine) have been used to prevent and treat influenza A virus infections for many years; however, resistance to these drugs has been widely reported in the world. To investigate the frequency and distribution of M2 gene mutations in adamantane-resistant influenza variants circulated in the world between 1902 and 2013, 31251 available M2 protein sequences from different HA-subtype influenza A viruses (H1–H17) were analyzed and adamantane resistance-associated mutations were compared (L26F, V27A, A30T, A30V, S31N, G34E, and L38F). We find that 45.2% (n = 14132) of influenza A (H1–H17) viruses circulating globally were resistant to adamantanes, and the vast majority of resistant viruses (95%) bear S31N mutations. Whereas, only about 1% have V27A mutations and other mutations (L26F, A30T, G34E, and L38F) were extremely rare (their prevalence appeared to be < 0.2%). Our results confirm that H1, H3, H5, H7, H9, and H17 subtype influenza A viruses exhibit high-level resistance to adamantanes. In contrast, the appearance of adamantane-resistant mutants in H2, H4, H6, H10, and H11 subtypes was rare. However, no adamantane resistance viruses were identified among other HA subtypes (H8, H12–H16). Our findings indicate that the frequency and distribution of adamantane-resistant influenza variants varied among different HA subtypes, host species, years of isolation, and geographical areas. This comprehensive study raises concerns about the increasing prevalence of adamantane-resistant influenza A viruses and highlights the importance of monitoring the emergence and worldwide spread of adamantane-resistant variants.

## Introduction

Influenza A virus is a common cause of respiratory infections, infecting humans, birds, pigs, horses, as well as other species [1]. Eighteen Hemagglutinin (HA, H1 to H18) and eleven Neuraminidase (NA, N1 to N11) subtypes of influenza viruses can cause epidemics and pandemics through antigenic drift and antigenic shift, respectively [1]. Considering the significant threat that influenza A viruses pose to global economy and human health, preparedness for a potential influenza pandemic is a global priority [2].

The primary means to protect humans against influenza virus infection is by vaccination. However, in the absence of an effective and widely available vaccine, protection from a pandemic and/or long-term endemicity would rely largely on the prophylactic and therapeutic properties of antiviral treatment. Therefore, effective anti-influenza drugs offer a valuable addition to the use of vaccines, particularly for those not caused by drug-resistant viruses [3]. Currently, two classes of anti-influenza drugs, adamantanes (amantadine and its methyl derivative rimantadine) and neuraminidase inhibitors (oseltamivir and zanamivir), are used for prophylaxis and treatment of influenza A infections [4]. These FDA-approved drugs are effective for influenza A viruses, including highly pathogenic H5N1 viruses [5]. Their therapeutic efficacy, however, has come into question with the emergence of drug-resistant viruses. Adamantane drugs, also known as M2 channel blockers, could block the ion channel formed by the M2 protein encoded by the M gene of influenza A viruses and inhibit early stages of virus replication [6]. As adamantane derivatives, amantadine (SymmetrelTM) and rimantadine (FlumadineTM) have been used successfully for influenza A virus infection for more than 30 years because of their wide availability and low cost [7, 8]. The effectiveness of prophylaxis with these drugs varied between 80% and 90%, and their use can reduce the duration of illness by 1.5 days if treatment is begun within 48 hours of infection [9]. However, the main drawbacks of ultilizing adamantane derivatives are that drug-resistant variants develop rapidly [10]. Presently, animal experiments and clinical studies have indicated the frequent occurrence of amantadine-resistant inﬂuenza variants after exposure to the drugs. During clinical use in humans, adamantane resistance emerged in about 30% of patients after 2–3 days of the treatment [11]. Moreover, drug-resistant variants can replicate as efficiently as sensitive ones, and they can also transmit efficiently from one individual to other individuals without apparent loss of pathogenicity [11]. Additionally, complete cross-resistance exists between amantadine and rimantadine because of the common mechanism of action [12]. To reduce the emergence and spread of drug-resistant influenza viruses, amantadine-oseltamivir combination chemotherapy has been evaluated and their synergistic antiviral effects have been confirmed [13]. Therefore, understanding the mechanism responsible for the emergence and spread of antiviral resistance is important for controlling seasonal and pandemic influenza.

After four decades of effective use of adamantanes in the prophylaxis and treatment of inﬂuenza, global resistance to these drugs has increased dramatically. In the period from 1991 to 1995, the global frequency of adamantane resistance among A (H3N2) viruses was as low as 0.8% [14]. However, in recent years, a significant worldwide increase in adamantane resistance has been reported for human H1N1 and H3N2 seasonal influenza viruses and H5N1 avian influenza viruses [15–17]. For example in China, the frequency of amantadine-resistant H1N1 variants has greatly increased from 28% during 2004–2005 to 72% in 2005–2006 [18]. Similarly, in Taiwan and Canada, the frequencies of adamantane-resistance raised from 0 in 2004–2005 to 24% and 33% in 2005–2006, respectively [18]. In the United States, resistance to adamantine among A (H3N2) viruses circulating had reached 92% in 2005 [19]. Specially, adamantane resistance had reached 100% during 2005–2006 in inﬂuenza A (H3N2) isolates from some Southeast Asian countries [18]. It is worth mentioned that the most recent H5N1 strains isolated from humans and poultry in Vietnam and Thailand show resistance to the adamantanes [20]. The rapid emergence and spread of adamantane-resistant variants of influenza H1N1, H3N2, recent H7N9 viruses from China, and H5N1 viruses have led to changes in CDC recommendations for the use of adamantanes in the control of influenza A virus infections [21].

The genetic basis for resistance to adamantanes has been well characterised and amino acid substitutions at residues L26, V27, A30, S31, and G34 within the transmembrane domain of the M2 protein are associated with adamantane resistance [12, 22]. Abed et al. constructed recombinant H1N1 influenza A viruses with the commonly observed L26F, V27A, A30T, S31N, G34E, and V27A/S31N mutations in the M2 gene by using reverse genetics, and showed all of these mutations are able to cause amantadine resistance [23]. So far, the known mutations that confer resistance against adamantanes are L26F, V27A, A30T, A30V, S31N, G34E and L38F in the M2 transmembrane region [6, 22]. Bright et al. found that out of 7000 inﬂuenza A isolates collected from countries in Africa, Asia, Europe, the Americas and Oceania between 1994 and 2005, the frequency of adamantane resistance mutations rose from 0.4% in 1994–1995 to 12.3% in 2003–2004 [16]. Most drug-resistant inﬂuenza viruses contain one of these amino acid changes, but variants with dual mutations have also been described [12]. In particular, the S31N substitution renders the virus highly resistant to adamantanes, and the incidence of clinical isolates bearing this mutation has jumped from a small percentage to approximately 97% in recent years [18, 19, 24]. Given the uncertainty of the therapeutic value about adamantanes, particular to a pandemic and/or long-term endemicity, it is necessary to study deeply whether resistance to adamantanes is prevalent in various inﬂuenza viruses in the world. In this present study, we analyzed the incidence rate, host distribution, temporal distribution, and geographic distribution of adamantane-resistance among H1–H17 subtype inﬂuenza A viruses isolated worldwide from 1902 to 2013, and also tried to clarify the possible reasons of high adamantane-resistance incidence.

## Methods

### Sequence data

All amino acid sequence data of the M2 proteins used in this study were obtained from the influenza virus sequence database available in GenBank (Influenza Virus Resource [http://www.ncbi.nlm.nih.gov/genomes/FLU/FLU.html], accessed on 8 June and 31 October 2013). Firstly, the shorter and identical sequences were collapsed using the web servers of Influenza Virus Resource in the National Center for Biotechnology Information (NCBI), and the remaining sequences were then utilized for further analysis. Secondly, a total of 31251 M2 protein sequences (nucleotide positions 1–27 and 716–979) were selected and downloaded representing the spectrum of influenza A diversity from 1902 to 2013, together with information about the subtype, host, location and year of isolation. Subtypes H1–H17 of influenza A virus from different host species, including human, avian, swine, equine, and canine viruses, were included. As a result, M2 protein sequences of 12579 H1, 539 H2, 9414 H3, 1001 H4, 2856 H5, 1208 H6, 1147 H7, 124 H8, 1151 H9, 548 H10, 421 H11, 133 H12, 74 H13, 11 H14, 7 H15, 35 H16, and 3 H17 influenza A viruses were used for the analysis. All GenBank accession numbers of adamantane resistant viruses were listed in the S1–S6 Tables through primary classification in host, subtype, and isolation location.

### Sequence Analysis

All of these sequence data were aligned by Clustal W (BioEdit version 7.0.5) (http://www.mbio.ncsu.edu/BioEdit/bioedit.html). Substitutions of residues Leu26, Val27, Ala30, Ser31, Gly34, His37, Leu38, and Trp41 in the transmembrane region of the M2 ion channel protein were used to screen amantadine-resistant mutants [12, 25]. The frequencies of the appearance of recognized adamantine resistance-associated amino acids (L26F, V27A, A30T, A30V, S31N, G34E, and L38F) were identified and tabulated. Among 31251 sequences, the number of strains with mutations was shown, and their detail information was listed in the S1–S6 Tables.

## Results

### The frequency of adamantane resistant influenza variants

A total of 31251 influenza A viruses were screened for the specific mutations known to correlate with resistance to adamantanes. Our analysis based on the M2 protein sequence data available in the GenBank showed a diversity in the frequency of adamantane resistant influenza variants among different HA subtypes, host species, years of isolation, and geographical areas (S1–S6 Tables). In the present study, a total of 14132 (45.22%) of the 31251 influenza A viruses contained one or two amino acid substitutions in the M2 protein known to cause resistance to adamantanes [12, 22]. The prevalence of adamantane resistance over the time period of the study is shown in Table 1. These results suggested that the frequency of emergence of drug-resistant strains varied among different HA subtypes. Among these isolates, we identified adamantane-resistant influenza variants from H1–H7, H9–H11, and H17 subtypes. However, analysis of viruses isolated from 1902 to 2013 did not reveal H8 and H12–H16 subtype influenza isolates with amino acid substitutions in the transmembrane region of M2 protein corresponding to resistance to adamantanes (Table 1). In other words, no adamantane resistant viruses were identified among the influenza H8, H12, H13, H14, H15, and H16 subtype. In contrast, adamantane-resistant strains frequently occurred among viruses of six HA subtypes with the frequencies of H1 (8777/12579, 69.78%), H3 (4107/9414, 43.63%), H5 (804/2856, 28.15%), H7 (144/1147, 12.55%), H9 (265/1151, 23.02%), and H17 (3/3, 100%). The remaining HA subtype influenza viruses were identified with <0.5% adamantane resistant variants except H6 subtype influenza viruses. Analysis of sequence data from H6 subtype strains indicated 2.07% (25/1208) resistant variants with M2 protein-associated mutations that would confer resistance to adamantanes [22]. Notably, the numbers of resistant variants differed among HA/NA subtype (Table 1). For example, a total of 8193 influenza A (H1N1) viruses and 583 influenza A (H1N2) isolates were found to be adamantane-resistant. Similarly, 4095 influenza A (H3N2), 7 influenza A (H3N1) and 5 influenza A (H3N8) viruses were found to contain mutations conferring resistance to adamantanes (Table 1). Our study further suggested that the frequency of emergence of adamantane-resistant strains varied among different HA or HA/NA subtypes.

**Table 1:** The frequencies of adamantane-resistant variants among all influenza A (H1-H17) viruses used in this study.

Viruses				The number of different HA/NA subtype resistant variants	Percentage ofresistant variants(%)^c^
HA subtypes	From GenBank	The number of resistant variants ^a^	Year of isolation^b^		
H1	12579	8777	1933–2013	H1N1(8193), H1N2(583)，H1(1)	69.78
H2	539	1	1968	H2N2(1)	0.19
H3	9414	4107	1968–2013	H3N2(4095), H3N1(7), H3N8(5)	43.63
H4	1001	3	2003, 2011	H4N2(2), H4N6(1)	0.30
H5	2856	804	1959–2013	H5N1(766), H5N2(26), H5N5(5), H5N3(3), H5(4)	28.15
H6	1208	25	1999–2013	H6N1(20), H6N2(3), H6N6(1), H6N8(1)	2.07
H7	1147	144	1902–2013	H7N9(65), H7N2(45), H7N7(31), H7N3(2), H7N1(1)	12.55
H8	124	0			0
H9	1151	265	1998–2013	H9N2(262), H9N8(1), H9(2)	23.02
H10	548	1	2012	H10N7(1)	0.18
H11	421	2	2007, 2011	H11N9(1), H11N3(1)	0.48
H12	133	0			0
H13	74	0			0
H14	11	0			0
H15	7	0			0
H16	35	0			0
H17	3	3	2009, 2010	H17N10(3)	100
Overall	31251	14132	1902–2013	H1-H17(14132)	45.22

^a^ adamantane resistance-associated mutations present in the M2 Protein of viruses were at positions L26F, V27A, A30T, A30V, S31N, G34E, and L38F that confer resistance to adamantanes.

^b^ Appearance time to adamantane resistant variants.

^c^ The frequency of adamantane resistant variants among different HA subtypes.

The frequencies of adamantane resistance mutations of viruses from influenza H1–H17 subtypes are also analyzed in this study. The critical residues conferring resistance to adamantanes were shown in Table 2. Results from our analyses indicated that the viruses with L26F, V27A, A30T, G34E, and L38F mutations were found sporadically. However, the majority of drug-resistant influenza variants contained point mutations resulting in a serine to-asparagine change at amino acid 31 (S31N) of the M2 protein that conferred adamantane resistance [22]. Among these resistant viruses, 96.7% (8490/8777) of the H1, 93.6% (3844/4107) of the H3, 66.7% (2/3) of the H4, 83.3% (670/804) of the H5, 92% (23/25) of the H6, 86.1% (124/144) of the H7, 87.5% (232/265) of the H9, 100% (1/1) of the H10, 50% (1/2) of the H11, and 100% (3/3) of the H17 strains demonstrated the S31N substitution (Table 2). Furthermore, influenza H1, H3 and H5 variants contained the L26F, V27A, A30T, L26F/S31N, and V27A/S31N adamantane resistant mutations (Table 2). These resistant strains possessed amino acid substitutions in the M2 protein at three positions–L26F, V27A, and S31N [22]. The G34E and G34E/S31N mutations emerged only in influenza A H1 viruses, whereas the L38F mutation only occurred in the influenza A H2 virus. In addition, the L38F/S31N and A30V/S31N mutations were only found in influenza A H3 viruses. Our study further revealed adamantane resistance mutations appeared across different HA subtypes.

**Table 2:** The frequency of adamantane resistance mutations of viruses from different influenza HA subtypes.

Adamantane-resistant mutation	The number of adamantane resistance mutations of viruses from different influenza HA subtypes										
	H1	H2	H3	H4	H5	H6	H7	H9	H10	H11	H17
L26F	2		8		4		2				
V27A	5		9		95		3	26		1	
A30T	2		2		4	1		6			
S31N	8490		3844	2	670	23	124	232	1	1	3
G34E	2										
L38F		1									
L26F /S31N	23		16	1	10			1			
V27A/S31N	250		224		21	1	15				
A30T/S31N	1		1								
A30V/S31N			2								
G34E/S31N	2										
L38F/S31N			1								
Overall	8777	1	4107	3	804	25	144	265	1	2	3

### Host distribution of adamantane resistant influenza variants

Adamantane resistance is caused by a single point mutation at the following amino acid positions in the transmembrane region of the M2 protein: 26, 27, 30, 31, 34, and 38. The known mutations that confer adamantane resistance are L26F, V27A, A30T, A30V, S31N, G34E and L38F [12, 22]. To better understand host distribution of adamantane resistance, we conducted the M2 protein sequence analyses focusing on positions 26, 27, 30, 31, 34, and 38 for amino acid substitutions associated with naturally occurring adamantane resistance. The numbers of adamantane resistant influenza variants with drug resistance mutation(s) among different hosts are shown in Table 3 and Table 4. Of those, adamantane resistant influenza A H1 and H3 variants mainly emerged in human and swine. In contrast, influenza A H5, H7 and H9 subtype viruses with adamantane resistance primarily occurred in avian. Particularly, influenza A H4, H6, H10, and H11 subtype adamantane resistant strains were only detected in avian species, whereas influenza H2 and H17 subtype adamantane resistant variants were only found in human and bat, respectively (Table 3). Adamantane-resistant mutations known as L26F, V27A, A30T, S31N, G34E, and L38F were detected at all six sites (positions 26, 27, 30, 31, 34, and 38) of M2 protein in human influenza viruses (Table 4). We also found influenza strains with double adamantane resistance mutations (L26F/S31N, V27A/S31N, A30T/S31N, A30V/S31N, G34E/S31N, and L38F/S31N) at positions 26 and 31, 27 and 31, 30 and 31, 31 and 34 or 31 and 38 in the human population. Adamantane-resistant mutations (L26F, V27A, A30T, S31N) were found at positions 26, 27, 30, and 31 in avian and swine influenza viruses. Double resistance mutations at positions 26 and 31 (L26F/S31N), 27 and 31 (V27A/S31N) were also found in these hosts (Table 4). However, No G34E resistant mutation at position 34 was detected in avian influenza viruses. Similarly, Avian and swine influenza viruses also had no L38F adamantane resistant mutation at position 38. Notably, adamantane resistance mutations in all hosts were detected most frequently at position 31 (S31N), followed by position 27 (V27A). The most common drug resistance mutation was S31N in human, avian, and swine influenza viruses with the frequencies of 98.7% (10813/10954), 88.1% (909/1032), and 77.4% (1595/2062), respectively. In addition, a small number of S31N adamantane resistance mutations were also detected in canine, cheetah, ferret, mink, cat, equine, bat, and environment influenza viruses (Table 4). In S1–S6 Tables we show more adamantane resistant variants carrying the S31N resistance mutation at position 31 in H3N2 and H1N1 human influenza viruses after 2003. Adamantane resistant strains of avian influenza H5N1 viruses have also been found more frequently since 2000. Our study further suggested that the frequency of emergence of adamantane-resistant strains varied among different hosts and adamantane resistance mutations appeared across different hosts.

**Table 3:** Host distribution of adamantane resistant influenza variants analyzed in this study among the different HA subtypes.

HA subtypes	The number of adamantane resistant influenza variants among different hosts													overall
	Human	Avian	Swine	Wild boar	Canine	Cheetah	Ferret	Mink	Cat	equine	bat	environment	unknown	
H1	7232	6	1525	1	2	2	4	1	4					8777
H2	1													1
H3	3568	10	525		2			2						4107
H4		3												3
H5	143	632	6						1			20	2	804
H6		25												25
H7	10	115										17	2	144
H9		238	6							1		20		265
H10		1												1
H11		2												2
H17											3			3
Overall	10954	1032	2062	1	4	2	4	3	5	1	3	57	4	14132

**Table 4:** Host distribution of adamantane-resistance mutations analyzed in this study.

Adamantane-resistant mutation	The number of adamantane resistant mutations from all of the HA subtypes among different hosts												
	Human	Avian	Swine	Wild boar	Canine	Cheetah	Ferret	Mink	Cat	equine	bat	environment	unknown
L26F	7	7	2										
V27A	63	68	5						1			1	1
A30T	2	12	1										
S31N	10813	909	1595		4	2	4	2	4	1	3	51	2
G34E	2												
L38F	1												
L26F /S31N	10	11	29									1	
V27A/S31N	50	25	429	1				1				4	1
A30T/S31N	2												
A30V/S31N	2												
G34E/S31N	1		1										
L38F/S31N	1												
Overall	10954	1032	2062	1	4	2	4	3	5	1	3	57	4

### Geographical distribution of adamantane-resistant influenza variants

To further understand the geographical distribution of adamantane resistance, the M2 protein sequences related to drug-resistant phenotypes were analyzed focusing solely on geographic location of influenza variants with adamantane-resistant mutations. The geographic analysis summarized in Table 5 indicated a substantially rising percentage of adamantane-resistant influenza viruses isolated from the world including specific countries in Africa, Asia, Europe, America, and Oceania. Adamantane resistant influenza A H1 variants were distributed widely in the world. Of these variants, 52.1% (4572/8777) distributed in several countries in America (United States, Canada, Mexico, and Nicaragua) and 26.4% (2315/8777) were detected in Asia (China, Singapore, Thailand, Hong Kong, South Korea, Cambodia, Viet Nam, Japan, and Taiwan). In addition, about one-fifth of the influenza H1 variants distributed in Europe (United Kingdom, Spain, Greece, Germany, Finland, Russia, and Netherlands) (Table 5). Adamantane resistant influenza A H3 variants were obtained mainly from 26 countries, showing a wide distribution across the world. Of those, 34.8% (1427/4107) isolates from Asia and 56.4% (2316/4107) variants from America were adamantane resistant. All influenza A H6 and H9 variants with adamantane resistance only distributed in Asia (China, Taiwan, and Hong Kong). For influenza A H5 adamantane resistance variants, a great number of them were distributed in Asia including China, Thailand, Cambodia, Viet Nam, and Indonesia, while a small number of them were found in Africa (Egypt). Importantly, the largest proportion of Asian drug-resistant avian influenza viruses of H5 and H9 subtypes occurred in China (Table 5). Influenza A H7 adamantane resistance variants distributed primarily in China of Asia and United States of America, with the highest frequency of resistance found in China (64.6%). Similarly, geographical analyses of adamantane-resistant mutations were also conducted in this study. The numbers of adamantane-resistant mutations from all of the HA subtypes are shown in Table 6. The L26F mutation distributed primarily in Hong Kong and New Zealand, the V27A mutation mainly distributed in China and Indonesia, and the A30T mutation distributed primarily in China. The S31N mutation distributed across the world (Table 6). This is the most common mutation known to confer resistance to adamantanes. In contrast, the G34E and L38F mutations only distributed in United States and South Korea, respectively. For double adamantane resistance mutations, the L26F/S31N mutations distributed mainly in Thailand, United Kingdom, and United States. The majority of the V27A/S31N mutations emerged in United States, Spain, South Korea, and Indonesia (Table 6). The A30T/S31N mutations were found in Nicaragua and Australia, and the G34E/S31N mutations occured in United Kingdom and Canada. The L38F/S31N and A30V/S31N mutations distributed only in Taiwan and United States, respectively. Our study further suggested adamantane resistance mutations appeared across different geographic regions and the frequency of emergence of adamantane-resistant strains varied among different geographical areas.

**Table 5:** Geographical distribution of adamantane-resistant influenza variants.

Geographiclocation	The number of adamantane-resistant variants from different HA subtypes					
	H1	H3	H5	H6	H7	H9
Africa						
Kenya	52	42				
Egypt			52			
Other Africa^a^	13	61	3			
Total Africa	65/8777(0.7%)	103/4107(2.5%)	55/804(6.8%)			
Asia						
China	662	532	119	4	93	124
Singapore	539	166				
Thailand	314	61	164			
Hong Kong	216	255	7			61
South Korea	64	27	1			2
Cambodia	39	30	56			
Viet Nam	70	91	249	3		8
Japan	102	51	1			2
Taiwan	135	129	4	18	2	
Indonesia			108			
Other Asia^b^	174	85	22		2	68
Total Asia	2315/8777(26.4%)	1427/4107(34.8%)	731/804(90.9%)	25/25(100%)	97/144(67.4%)	265/265(100%)
Europe						
United Kingdom	422	10				
Spain	389	15				
Greece	122					
Germany	101	48				
Finland	128	2				
Russia	105					
Netherlands	45	15				
Other Europe^c^	355	99	1		2	
Total Europe	1667/8777(19.0%)	189/4107(4.6%)	1/804(0.1%)		2/144(1.4%)	
America						
United States	3644	2122	1		45	
Canada	335	11				
Mexico	174	35	13			
Nicaragua	141	111	0			
Other America^d^	278	37	3			
Total America	4572/8777(52.1%)	2316/4107(56.4%)	17/804(2.1%)		45/144(31.3%)	
Oceania						
Australia	150	40				
New Zealand	8	32				
Total Oceania	158/8777(1.8%)	72/4107(1.8%)				
Overall	8777	4107	804	25	144	265

^a^ Ethiopia, Mali,Nigeria, Senegal, South Africa, Uganda.

^b^ Bangladesh, India, Iran, Israel, Kazakhstan, Kuwait, Kyrgyzstan, Laos, Macau, Malaysia, Mongolia, Myanmar, Pakistan, Philippines, Qatar, Saudi Arabia, Sri Lanka,Turkey, Turkmenistan, United Arab Emirates.

^c^ Belarus, Belgium, Czech Republic, Denmark, Estonia, France, Hungary, Ireland, Italy, Norway, Poland, Portugal, Serbia, Sweden, Switzerland.

^d^ Argentina, Bolivia, Brazil, Chile, Costa Rica, Cuba, Dominican Republic, Ecuador, EI Salvador, Guam, Guatemala, Haiti, Peru, Puerto Rico.

**Table 6:** Geographical distribution of adamantane-resistant mutations analyzed.

Geographiclocation	The number of adamantane-resistant mutations from all of the HA subtypes											
	L26F	V27A	A30T	S31N	G34E	L38F	L26F/ S31N	V27A/S31N	A30T/S31N	A30V/S31N	G34E/S31N	L38F/S31N
Africa												
Kenya				94								
Egypt				52								
Other Africa^a^				77			1					
Total Africa				223			1					
Asia												
China	1	35	8	1483			2	8				
Singapore				705								
Thailand			2	531			6					
Hong Kong	4	5	1	529								
South Korea		2	1	76		1		15				
Viet Nam		1		414				8				
Japan			1	151			1	2				
Taiwan				285			1	1				1
Indonesia		70		28				10				
Other Asia^b^	5	16		437			3	5				
Total Asia	10	129	13	4639		1	13	49				1
Europe												
United Kingdom		1		423			6	1			1	
Spain		4		360				40				
Greece				122								
Germany				137				12				
Finland				128				2				
Russia				152			1					
Netherlands				60								
Other Europe^c^				379			9	21				
Total Europe		5		1761			16	76			1	
America												
United States	1	5	1	5401	2		17	378		2		
Canada	1			342				1			1	
Mexico	1			221								
Nicaragua				251					1			
Other America^d^				330			4	3				
Total America	3	5	1	6545	2		21	382	1	2	1	
Oceania												
Australia				186				4	1			
New Zealand	3		1	36								
Total Oceania	3		1	222				4	1			
Overall	16	139	15	13390	2	1	51	511	2	2	2	1

### Temporal distribution of adamantane resistant influenza variants

To better understand time distribution of adamantane resistant influenza variants, the M2 protein sequence analyses were conducted focusing attention on years of isolation of influenza viruses with adamantane resistance. The numbers and frequencies of adamantane resistant variants from H1N1, H3N2, H5N1, H6, H7, and H9N2 subtype influenza viruses among different years of isolation are given in Table 7–12. The trend for overall data showed a considerable increase. And the frequency of emergence of adamantane-resistant variants from different HA subtypes varied among different years of isolation during 2001–2013. For H1 subtype, temporal distribution of adamantane-resistant influenza H1N1 variants during 1933–2013 revealed substantial increases in rates for USA, China, Singapore, United Kingdom, Spain, Canada, Thailand, Hong Kong, Mexico, Australia, Nicaragua, Finland, Greece, Russia and Taiwan (Table 7). Trends of rate changes from these countries were not considerable before the spikes occurred in 2009, which indicate the existence of such spikes and their considerable effects on the overall trends. As shown in Table 7, the highest level of adamantane resistance was continually seen during 2009–2013 at 100% in many countries (e.g. Singapore, Spain, Portugal, Nicaragua, Chile). For H3 subtype, temporal distribution of adamantane-resistant variants from H3N2 influenza viruses during 1968–2013 showed substantial increases in rates from many countries and regions across the five continents: USA, China, Hong Kong, Singapore, Taiwan, Nicaragua, Vietnam, Thailand, Uganda, Japan, Russia, Germany, Kenya, and Australia (Table 8). The frequency of adamantane-resistant H3N2 influenza viruses has increased sharply since 2003, particularly in China. The proportion of isolates resistant to adamantanes continued to increase during 2005–2006. Specifically, isolates collected during this period in Vietnam, Spain, Mexico, Cuba, and New Zealand exhibited 100% drug resistance. During 2003–2009, resistants frequency increased considerably in mainland China, Hong Kong, Taiwan and Thailand. During 2005–2013, there were substantial increases in the rates in USA and Singapore. In contrast, no adamantane resistance was observed in United Kingdom during this period. During 2008–2010, the overall rate change in Kenya, Uganda, Russia, Netherlands, Mexico, and Japan was considerable.

**Table 7:** Temporal distribution of adamantane-resistant influenza variants from H1N1 subtypes viruses during 1933–2013.

Geographiclocation	The number and frequency of adamantane-resistant variants from H1N1 subtypes influenza viruses													
	By 2000	2001	2002	2003	2004	2005	2006	2007	2008	2009	2010	2011	2012	2013
Kenya	0/0	0/0	0/0	0/0	0/0	0/0	0/0	0/0	0/10	50/50 (100%)	0/0	0/0	0/0	2/3 (66.7%)
China	2/172 (1.1%)	2/35 (5.7%)	0/18	0/2	0/2	26/36 (72.2%)	96/112 (85.7%)	76/79 (96.2%)	80/103 (77.7%)	251/349 (71.9%)	68/70 (97.1%)	45/48 (93.8%)	3/3 (100%)	0/0
Singapore	0/1	0/0	0/0	0/0	0/0	0/0	0/0	1/1 (100%)	0/0	310/311 (99.7%)	179/179 (100%)	49/49 (100%)	0/0	0/0
Thailand	1/1 (100%)	0/0	0/0	1/2 (50.0%)	2/2 (100%)	2/2 (100%)	6/37 (16.2%)	1/4 (25.0%)	1/11 (9.1%)	87/94 (92.6%)	201/201 (100%)	7/8 (87.5%)	0/0	0/0
Hong Kong	0/78	3/17(17.6%)	1/5(20.0%)	3/5(60.0%)	4/5(80.0%)	6/10(60.0%)	8/11(72.7%)	5/7(71.4%)	9/13(69.2%)	163/183(89.1%)	1/3(33.3%)	4/5(80.0%)	0/0	0/0
South Korea	0/0	0/0	0/0	0/0	0/3	0/3	0/2	1/2 (50.0%)	0/0	45/48 (93.8%)	7/8 (87.5%)	3/5 (60.0%)	3/4 (75.0%)	0/0
Viet Nam	0/0	0/8	0/8	0/34	0/0	0/7	1/18 (5.6%)	0/0	12/39 (30.8%)	56/56 (100%)	0/0	0/0	0/0	0/0
Japan	0/3	0/2	0/7	0/0	0/0	0/8	0/6	3/5 (60.0%)	10/26 (38.5%)	79/93 (84.9%)	2/2 (100%)	0/0	0/0	0/0
Taiwan	0/21	1/2 (50.0%)	2/14 (14.3%)	0/2	0/4	1/6 (16.7%)	15/52 (28.8%)	9/18 (50.0%)	5/19 (26.3%)	43/57 (75.4%)	7/9 (77.8%)	22/32 (68.8%)	0/0	0/0
India	0/0	0/0	0/0	0/0	0/0	0/0	0/0	0/4	0/0	29/31 (93.5%)	9/12 (75.0%)	4/4 (100%)	8/8 (100%)	3/3 (100%)
Denmark	1/2 (50.0%)	0/2	0/0	0/0	0/1	0/2	0/1	0/2	0/11	39/39 (100%)	1/1 (100%)	16/16 (100%)	0/0	0/0
United Kingdom	36/44 (56.3%)	2/2 (100%)	1/1 (100%)	1/1 (100%)	1/2 (50.0%)	2/2 (100%)	3/7 (42.9%)	1/4 (25.0%)	0/8	302/304 (99.3%)	49/50 (98.0%)	7/8 (87.5%)	0/0	0/0
Spain	1/1 (100%)	0/0	0/0	2/2 (100%)	1/1 (100%)	0/0	0/0	3/3 (100%)	2/2 (100%)	367/367 (100%)	2/2 (100%)	5/5 (100%)	0/0	0/0
Greece	0/0	0/0	0/0	0/0	0/0	0/0	0/0	0/0	0/0	90/90 (100%)	19/19 (100%)	13/14 (92.9%)	0/0	0/0
Germany	8/18 (44.4%)	1/1 (100%)	0/0	3/3 (100%)	3/4 (75.0%)	3/18 (16.7%)	1/11 (9.1%)	1/1 (100%)	0/2	48/48 (100%)	5/5 (100%)	9/11 (81.8%)	0/0	0/0
Finland	0/0	0/0	0/0	0/0	0/0	0/0	0/0	0/0	0/0	9/10 (90.0%)	0/0	1/1 (100%)	7/7 (100%)	111/111 (100%)
Russia	1/6 (16.7%)	0/0	0/0	0/0	0/0	0/0	1/1 (100%)	0/0	0/0	64/64 (100%)	6/7 (85.7%)	29/31 (93.5%)	4/6 (66.7%)	0/0
Portugal	0/0	0/0	0/0	0/0	0/0	0/0	0/0	0/0	0/0	53/53 (100%)	4/4 (100%)	0/0	0/0	0/0
France	6/13 (46.2%)	0/3	1/3 (33.3%)	0/0	1/1 (100%)	0/0	0/1	0/1	0/0	44/47 (93.6%)	3/3 (100%)	0/0	0/0	0/0
United States	12/287 (4.2%)	0/36	1/34 (2.9%)	0/34	0/1	0/10	3/29 (10.3%)	12/436 (2.8%)	7/203 (3.4%)	1947/2303 (84.5%)	334/401 (83.3%)	322/399 (80.7%)	390/449 (86.9%)	185/208 (88.9%)
Canada	0/25	0/0	0/9	0/4	2/7 (28.6%)	0/7	0/4	0/7	0/2	319/373 (85.5%)	10/40 (25.0%)	0/1	1/1 (100%)	0/0
Mexico	0/0	0/0	0/0	0/0	0/0	0/0	0/0	0/1	0/6	157/165 (95.2%)	12/19 (63.2%)	1/1 (100%)	4/4 (100%)	0/0
Nicaragua	0/0	0/0	0/0	0/0	0/0	0/0	0/0	0/0	0/37	137/137 (100%)	4/4 (100%)	0/0	0/0	0/0
Chile	0/3	0/0	0/0	0/0	0/0	0/0	0/0	0/0	0/0	53/53 (100%)	23/23 (100%)	0/0	0/0	0/0
Argentina	0/0	0/0	0/0	0/0	0/0	0/0	0/0	0/0	0/1	68/69 (98.6%)	1/1 (100%)	0/0	4/5 (80.0%)	0/0
Australia	3/24	1/5	0/0	0/0	0/1	0/6	1/2 (50.0%)	1/11 (9.1%)	0/0	70/71 (98.6%)	70/71 (98.6%)	4/4 (100%)	0/0	0/0

**Table 8:** Temporal distribution of adamantane-resistant influenza variants from H3N2 subtypes viruses during 1968–2013.

Geographiclocation	The number and frequency of adamantane-resistant variants from H3N2 subtypes influenza viruses													
	By 2000	2001	2002	2003	2004	2005	2006	2007	2008	2009	2010	2011	2012	2013
Kenya	0/0	0/0	0/0	0/0	0/0	0/0	0/1	0/0	30/30 (100%)	0/0	12/12 (100%)	0/0	0/0	0/0
Uganda	0/0	0/0	0/0	0/0	0/0	0/0	0/0	0/0	50/50 (100%)	9/9 (100%)	0/0	0/0	0/0	0/0
China	3/130 (2.3%)	1/24 (4.2%)	2/58 (3.5%)	41/70 (58.6%)	30/48 (62.5%)	44/108 (40.7%)	100/109 (91.7%)	179/187 (95.7%)	30/31 (96.8%)	65/73 (89.0%)	4/20 (20.0%)	0/8	0/0	0/0
Singapore	0/9	0/0	0/1	0/12	0/0	1/2(50.0%)	0/0	1/1(100%)	0/0	31/31(100%)	34/34(100%)	98/98(100%)	0/0	1/1(100%)
Thailand	0/5	0/0	3/3 (100%)	1/1 (100%)	3/4 (75.0%)	3/4 (75.0%)	2/8 (25.0%)	1/14 (7.1%)	16/17 (94.1%)	18/18 (100%)	3/3 (100%)	7/7 (100%)	4/4 (100%)	0/0
Hong Kong	13/327 (4.0%)	2/63 (3.2%)	2/65 (3.1%)	6/54 (11.1%)	48/96 (50%)	73/85 (85.9%)	10/14 (71.4%)	0/0	0/0	99/99 (100%)	0/0	2/2 (100%)	0/0	0/0
South Korea	0/0	0/0	0/0	0/2	1/11 (9.1%)	0/11	0/9	3/30 (10.0%)	3/14 (21.4%)	3/4 (75.0%)	0/11	2/5 (40.0%)	10/11 (90.9%)	0/0
Cambodia	0/0	0/0	0/0	0/0	0/0	1/3 (33.3%)	0/8	3/3 (100%)	11/11 (100%)	10/10 (100%)	0/0	5/5 (100%)	0/0	0/0
Viet Nam	0/0	0/1	0/6	0/14	1/29 (3.4%)	25/35 (71.4%)	1/1 (100%)	41/74 (55,4%)	12/12 (100%)	0/0	7/7 (100%)	1/1 (100%)	0/3	0/0
Japan	2/41 (4.9%)	0/0	0/2	0/0	0/3	10/15 (66.7%)	21/27 (77.8%)	3/7 (42.9%)	4/5 (80.0%)	4/4 (100%)	7/7 (100%)	0/0	0/0	0/0
Taiwan	1/34 (2.9%)	0/5	0/8	5/21 (23.8%)	12/41 (29.3%)	24/34 (70.6%)	17/19 (89.5%)	32/38 (84.2%)	13/13 (100%)	22/22 (100%)	3/4 (75.0%)	0/0	0/0	0/0
Malaysia	0/46	0/0	0/1	0/9	1/18 (5.6%)	7/9 (77.8%)	5/12 (41.7%)	17/19 (89.5%)	7/7 (100%)	2/2 (100%)	0/0	0/0	0/0	0/0
Philippines	0/8	0/0	0/0	0/0	0/0	1/2 (50.0%)	0/0	1/3 (33.3%)	0/0	0/0	7/7 (100%)	1/1 (100%)	2/2 (100%)	0/0
United Kingdom	6/23 (26.1%)	0/0	0/0	2/77 (2.6%)	0/0	0/0	0/0	0/0	0/0	0/0	0/0	0/0	0/0	0/0
Spain	0/10	1/1 (100%)	2/2 (100%)	0/0	1/1 (100%)	4/4 (100%)	0/0	3/3 (100%)	0/0	0/0	3/3 (100%)	1/1 (100%)	0/0	0/0
Germany	8/20 (40.0%)	1/1 (100%)	0/15	5/20 (25.0%)	4/19 (21.1%)	8/19 (42.1%)	22/24 (91.7%)	0/0	0/0	0/0	0/0	0/0	0/0	0/0
Russia	5/14 (35.7%)	0/0	0/0	0/7	0/0	0/0	0/0	0/0	2/2 (100%)	4/4 (100%)	1/1 (100%)	4/5 (80.0%)	32/32 (100%)	0/0
Netherlands	1/102 (1.0%)	0/3	0/2	0/8	0/1	1/7 (14.3%)	5/8 (62.5%)	2/5 (40.0%)	1/1 (100%)	2/2 (100%)	2/2 (100%)	1/1 (100%)	0/0	0/0
Romania	0/0	0/0	0/0	0/0	0/0	0/0	0/0	0/0	0/0	23/23 (100%)	0/0	0/0	0/0	0/0
United States	10/559 (1.8%)	0/15	0/79	2/114 (1.8%)	1/85 (1.2%)	18/84 (21.4%)	38/54 (70.4%)	128/153 (83.7%)	256/274 (93.4%)	319/374 (85.3%)	176/292 (60.3%)	439/472 (93.0%)	569/614 (92.7%)	164/176 (93.2%)
Canada	1/4 (25.0%)	0/0	0/0	0/3	0/2	0/10	0/9	0/7	0/1	0/11	6/6 (100%)	4/10 (40.0%)	0/0	0/0
Mexico	0/0	0/0	0/0	1/7 (14.3%)	0/0	5/5 (100%)	1/1 (100%)	0/0	11/11 (100%)	2/2 (100%)	15/18 (83.3%)	0/0	0/0	0/0
Nicaragua	0/0	0/0	0/0	0/0	0/0	0/0	0/0	36/36 (100%)	0/0	0/0	73/73 (100%)	2/2 (100%)	0/0	0/0
Cuba	0/0	0/0	0/0	0/0	0/0	0/0	1/1 (100%)	6/6 (100%)	0/0	2/2 (100%)	0/0	15/15 (100%)	0/0	0/0
Australia	4/93 (4.3%)	0/14	0/23	1/34 (2.9%)	0/26	13/50 (26.0%)	0/2	9/10 (90.0%)	0/0	9/9 (100%)	4/4 (100%)	0/0	0/0	0/0
New Zealand	1/78 (1.3%)	0/23	3/90 (3.3%)	0/88	2/114 (1.8%)	24/77 (31.2%)	1/1 (100%)	1/1 (100%)	0/0	0/0	0/0	0/0	0/0	0/0

**Table 9:** Temporal distribution of adamantane-resistant influenza variants from H5N1 subtype viruses during 1959–2013.

Geographiclocation	The number and frequency of adamantane-resistant variants from H5N1 subtype influenza viruses													
	By 2000	2001	2002	2003	2004	2005	2006	2007	2008	2009	2010	2011	2012	2013
Egypt	0/10	0/0	0/0	0/0	0/0	0/0	0/1	2/16 (12.5%)	18/23 (78.3%)	13/16 (81.3%)	15/17 (88.2%)	1/10 (10.0%)	0/2	0/0
China	0/10	2/18 (11.1%)	7/32 (21.9%)	15/55 (27.3%)	8/55 (14.5%)	12/146 (8.2%)	28/119(23.5%)	7/32 (21.9%)	11/20 (55.0%)	11/25 (44.0%)	0/3	6/21 (28.6%)	1/1 (100%)	0/0
Thailand	0/0	1/1 (100%)	0/0	3/3 (100%)	85/85 (100%)	37/37 (100%)	11/11 (100%)	6/7 (85.7%)	18/18 (100%)	0/0	3/3 (100%)	0/0	0/0	0/0
Hong Kong	0/44	0/6	2/15 (13.3%)	2/7 (28.6%)	0/1	0/0	0/18	1/27 (3.7%)	2/6 (33.3%)	0/0	0/2	0/3	0/0	0/0
South Korea	0/1	0/0	0/0	0/0	0/1	0/0	0/9	0/0	0/6	0/4	0/7	1/25 (4.0%)	0/0	0/0
Cambodia	0/0	0/0	0/0	0/0	1/1 (100%)	11/11 (100%)	13/13 (100%)	3/3 (100%)	4/4 (100%)	2/2 (100%)	7/7 (100%)	14/14 (100%)	1/1 (100%)	0/0
Viet Nam	0/74	0/0	0/0	17/17 (100%)	84/84 (100%)	61/81 (75.3%)	6/6 (100%)	59/65 (90.8%)	9/17 (52.9%)	4/11 (36.4%)	0/2	2/5 (40.0%)	7/19 (36.8%)	0/12
Indonesia	0/56	0/0	0/0	5/9 (55.6%)	2/9 (22.2%)	17/24 (70.8%)	58/76 (76.3%)	21/23 (91.3%)	5/5 (100%)	0/0	0/0	0/0	0/0	0/0
Laos	0/0	0/0	0/0	0/0	1/1 (100%)	0/0	0/6	1/22 (4.5%)	5/6 (83.3%)	0/0	0/20	0/0	0/0	0/0
India	0/0	0/0	0/0	0/0	0/0	0/0	3/8 (37.5%)	0/1	0/30	0/10	3/3 (100%)	0/8	0/0	0/0
Bangladesh	0/0	0/0	0/0	0/0	0/0	0/0	0/0	0/1	0/1	0/0	1/5 (20.0%)	1/4 (25.0%)	2/17 (11.8%)	0/0
Saudi Arabia	0/0	0/0	0/0	0/0	0/0	0/1	0/0	2/3 (66.7%)	0/0	0/0	0/0	0/0	0/0	0/0
Iran	0/0	0/0	0/0	0/0	0/0	0/0	0/1	0/0	1/1 (100%)	0/0	0/0	0/0	0/0	0/0
Malaysia	0/0	0/0	0/0	0/0	0/0	0/0	0/0	1/1 (100%)	0/0	0/0	0/0	0/0	0/0	0/0
Israel	0/0	0/0	0/0	0/0	0/0	0/0	0/4	0/0	1/1 (100%)	0/0	0/0	0/0	0/0	0/0
Elsalvador	0/0	1/1 (100%)	0/0	0/0	0/0	0/0	0/0	0/0	0/0	0/0	0/0	0/0	0/0	0/0
Belgium	0/0	0/0	0/0	0/0	1/1 (100%)	0/0	0/0	0/0	0/0	0/0	0/0	0/0	0/0	0/0

**Table 10:** Temporal distribution of adamantane-resistant influenza variants from H6 subtypes viruses during 1999–2013.

Geographiclocation	The number and frequency of adamantane-resistant variants from H6 subtypes influenza viruses													
	By 2000	2001	2002	2003	2004	2005	2006	2007	2008	2009	2010	2011	2012	2013
China (H6N1)	0/4	0/5	0/7	0/15	1/40 (2.5%)	1/15(6.7%)	0/3	0/1	0/1	0/0	0/1	0/0	0/0	0/0
Taiwan (H6N1)	2/15 (13.3%)	1/4 (25.0%)	1/9 (11.1%)	1/4 (25.0%)	3/3 (100%)	1/2 (50.0%)	0/0	0/0	0/0	6/6(100%)	3/3(100%)	0/0	0/0	0/0
Viet Nam (H6N2)	0/0	0/0	0/0	0/0	0/0	0/0	0/0	0/1	0/0	0/0	0/0	0/0	2/4 (50.0%)	1/1(100%)
China (H6N6)	0/13	0/0	0/0	0/0	0/0	0/0	0/36	0/49	1/1(100%)	0/12	0/10	0/1	0/1	0/0
China (H6N8)	0/6	0/0	0/1	0/0	1/1(100%)	0/0	0/2	0/5	0/0	0/0	0/1	0/0	0/0	0/0

**Table 11:** Temporal distribution of adamantane-resistant influenza variants from H7 subtypes viruses during 1902–2013.

Geographiclocation	The number and frequency of adamantane-resistant variants from H7 subtypes influenza viruses													
	By 2000	2001	2002	2003	2004	2005	2006	2007	2008	2009	2010	2011	2012	2013
China (H7N9)	0/0	0/0	0/0	0/0	0/0	0/0	0/0	0/0	0/0	0/11	0/0	0/0	0/0	63/63 (100%)
Taiwan (H7N9)	0/0	0/0	0/0	0/0	0/0	0/0	0/0	0/0	0/0	0/0	0/0	0/0	0/0	2/2 (100%)
China (H7N7)	0/0	0/0	0/0	0/7	0/0	0/0	0/0	0/0	0/4	0/3	0/5	0/0	0/2	30/32 (93.8%)
Italy (H7N7)	1/3 (33.3%)	0/0	0/0	0/0	0/0	0/1	0/2	0/0	0/0	0/0	0/0	0/0	0/0	0/0
China (H7N6)	0/0	0/0	0/0	0/0	0/0	0/0	0/0	0/1	0/0	0/2	0/0	0/0	0/0	1/1(100%)
Pakistan (H7N3)	0/15	0/1	0/0	0/2	2/4 (50.0%)	0/0	0/0	0/0	0/0	0/0	0/0	0/0	0/0	0/0
USA (H7N2)	11/99 (11.1%)	15/32 (46.9%)	1/17 (5.9%)	5/17 (29.4%)	0/3	13/161 (8.1%)	0/33	0/1	0/1	0/0	0/0	0/0	0/0	0/0
Italy (H7N1)	0/53	0/2	1/1(100%)	0/0	0/0	0/0	0/0	0/0	0/0	0/0	0/0	0/0	0/0	0/0

**Table 12:** Temporal distribution of adamantane-resistant influenza variants from H9N2 subtypes viruses during 1998–2013.

Geographiclocation	The number and frequency of adamantane-resistant variants from H9N2 subtypes influenza viruses													
	By 2000	2001	2002	2003	2004	2005	2006	2007	2008	2009	2010	2011	2012	2013
China	27/78 (34.6%)	2/43 (4.7%)	5/41 (12.2%)	5/47 (10.6%)	6/65 (9.2%)	6/78 (7.7%)	3/10 (30.0%)	4/37 (10.8%)	9/28 (32.1%)	20/23 (87.0%)	12/13 (92.3%)	16/28 (57.1%)	5/7 (71.4%)	2/2 (100%)
Hong Kong	1/24 (4.2%)	0/1	0/3	4/32 (12.5%)	0/0	3/7 (42.9%)	5/9 (55.6%)	1/16 (6.3%)	6/12 (50.0%)	0/8	8/13 (61.5%)	17/18 (94.4%)	16/16 (100%)	0/0
South Korea	0/12	0/2	0/3	0/5	0/12	0/13	0/13	0/7	1/8 (12.5%)	1/24 (4.2%)	0/2	0/2	0/0	0/0
Viet Nam	0/0	0/0	0/0	0/0	0/0	0/0	0/1	0/0	0/0	3/4 (75.0%)	0/0	0/0	2/3 (66.7%)	2/2 (100%)
Japan	0/5	1/8 (12.5%)	1/2 (50.0%)	0/0	0/0	0/0	0/0	0/0	0/0	0/0	0/0	0/0	0/0	0/0
Bangladesh	1/1 (100%)	0/0	0/0	0/0	0/0	0/0	0/1	0/0	0/0	4/7 (57.1%)	15/15 (100%)	21/22 (95.5%)	0/0	0/0
Iran	1/6 (16.7%)	0/1	0/0	0/1	0/0	1/1 (100%)	1/2 (50.0%)	2/3 (66.7%)	0/1	2/2 (100%)	1/1 (100%)	0/0	2/4 (50.0%)	0/0
Israel	0/17	0/1	0/0	0/4	0/3	3/7 (42.9%)	0/4	3/13 (23.1%)	0/11	0/2	0/5	0/0	0/0	0/0
United Arab	3/3 (100%)	3/3 (100%)	1/2 (50.0%)	1/1 (100%)	0/0	0/0	0/0	0/0	0/0	0/0	0/0	3/4 (75.0%)	0/0	0/0

For the H5 subtype, temporal distribution of adamantane-resistant influenza H5N1 variants during 1959–2013 indicated considerable rate changes from six distinctly important countries including China, Thailand, Cambodia, Vietnam, and Indonesia of Asia and Egypt of Africa. Furthermore, rates among these countries were also considerably different (Table 9). For instance, the frequency of H5N1 adamantane-resistant viruses obtained from Indonesia began increasing in 2003, with a considerable spike (100%) between 2007 and 2008. This trend of having spikes was followed by an increasing frequency in Thailand and Vietnam in 2003. Notably, the percentage of adamantane resistant H5N1 viruses gathered from Cambodia is particularly high and consistently reached 100% during 2004–2012. For H6 subtype, temporal distribution of adamantane-resistant H6 influenza variants during 1999–2013 suggested that H6N1 and H6N2 mutants with considerable rate changes emerged mainly in Taiwan and Vietnam, respectively (as shown in Table 10). No H6N2 resistant viruses were detected in samples collected from China. For H7 subtype, temporal distribution of adamantane-resistant H7 influenza variants during 1902–2013 suggested that H7N9, H7N7, and H7N6 mutants with considerable rate changes emerged mainly in China in 2013, whereas H7N3, H7N2, and H7N1 mutants were respectively detected in Pakistan, USA, and Italy by 2005 (as shown in Table 11). For H9 subtype, temporal distribution of H9N2 adamantane resistant viruses during 1998–2013 displayed considerable changes in rates in Asia for specific countries or areas such as China, Hong Kong, Bangladesh, and United Arab (Table 12). In 2009, one or more drug-resistant virus was identified in five countries: China (20/23, 87.0%), South Korea (1/24, 4.2%), Vietnam (3/4, 75.0%), Bangladesh (4/7, 57.1%), and Iran (2/2, 100%). However, no resistance was found in Hong Kong, Japan, Israel, and United Arab in the same period. By comparison, the frequency of resistant H9N2 variants isolated from China was consistently near 10% during 2002–2005.

In conclusion, our study further suggested the frequency of emergence of adamantane-resistant influenza strains varied among different years of isolation.

## Discussion

Anti-influenza drugs play an important role in a comprehensive approach on controlling influenza A virus infections. Adamantanes had been excellent anti-influenza medicines until the recent emergence of resistant viruses. In this study, 31251 different subtype influenza A viruses (H1–H17) isolated in the world from 1902 to 2013 were assessed for resistance to adamantanes. On the basis of M2 protein sequence analysis, our study reveals a continuing worldwide increase in adamantane resistance, and suggests that the frequency of emergence of drug-resistant influenza variants varied among different HA subtypes, host species, years of isolation, and geographical areas. This study, which evaluates the frequency and distribution of 14132 adamantane-resistant influenza viruses obtained worldwide, is the largest and most comprehensive report on adamantane resistance to date.

The frequency of resistance to adamantanes among circulating influenza A viruses has dramatically increased over the past few years [16]. In this study we confirm that H1, H3, H5, H7, H9, and H17 subtype influenza A viruses with the specific resistance-associated mutations in their M2 genes exhibited high-level resistance to adamantanes. In contrast, the appearance of H2, H4, H6, H10, and H11 adamantane-resistant mutants was a rare event. However, no adamantane resistance viruses were identified among other HA subtypes (H8, H12–H16) (Table 1). Our findings extend those reported in 2005 [16] and 2006 [19] and document a continuous and proportional increase in influenza A (H1, H3, H5, H7, H9) variants showing resistance to adamantanes. The global incidence of adamantane resistance among A (H3N2) has increased dramatically. This escalating trend in circulating H3N2 drug-resistant variants was first observed among viruses isolated from Asia in 2000 and then in other regions of the world in 2005 (Table 8). A significant increase in resistance was also detected in many countries throughout the world among A (H1N1) viruses collected during 2009–2012 (Table 7). Although adamantane resistant H5N1 variants are present in Asia, their distribution appeared to be largely limited to Thailand, Cambodia, and Vietnam, It is worth noting that most H5N1 viruses from Indonesia and China are sensitive to adamantine [5] (Table 9). The apparent geographical disparity in the susceptibility of H5N1 isolates to adamantane is unexplained. Similarly, H9N2 viruses isolated from Asian countries including China, Hong Kong, Bangladesh, and United Arab show significant increases in drug-resistance frequencies (Table 12). Particularlly, H7N9 resistant mutants only distributed in mainland China and Taiwan of Asia also show a significant increase in the incidence (100%) of adamantane resistance in 2003 (Table 11). Our findings that epidemic and pandemic strains of influenza were identified first in Asia accord with the results from other previous studies [26].

Adamantane resistance in the influenza A virus is associated with six amino acid substitutions in the M2 protein according to previous reports [16]. The known mutations that confer adamantane resistance are L26F, V27A, A30T (A30V), S31N, G34E, and L38F [12, 22]. It was reported that mutated viruses may either lose the ability to bind M2 ion channel blockers, as with the S31N or A30T amino acid substitutions [27], or bind the blockers but retain M2 function, as with amino acid replacements L26F or V27A at residue 26 or 27 [27]. In our study described here, the most common adamantane resistance mutation was S31N in influenza A viruses from human, avian, and swine (Table 4). Most adamantane-resistant influenza variants (95%) bear S→N amino acid mutations at position 31 of the M2 protein, whereas only about 1% have V→A mutations at aa 27. Drug-resistance mutations at other amino acid positions (L26F, A30T, G34E, and L38F) are extremely rare (their prevalence appeared to be < 0.2%) (Table 2). Among these mutations, the S31N is the most frequently reported [14, 19, 28], suggesting that variants containing the S31N substitution might possess a significant advantage on viral replication or transmission, leading to more efficient circulation. Additionally, the high levels of the dual L26I and S31N resistance mutations mainly detected in H5N1 variants obtained from Thailand, Vietnam, and Cambodia indicate that viruses carrying this dual motif are stably selected (S3 Table). Given that the dual L26I and S31N motif are identified firstly in China in 2002 in our study, it appears that China might have been the location of introduction or generation of viruses. Our results further suggest that M2 drug-resistant mutations could have occurred spontaneously before these drugs were developed, implying that greater caution is needed in the use of adamantanes.

It is known that influenza viruses resistant to amantadine and rimantadine can emerge quickly when these drugs are being used to control influenza outbreaks [11, 29]. Adamantanes have been considered first-line drugs for the prophylaxis and treatment of influenza A virus infections. The widespread use of adamantanes has been associated with the rapid emergence of resistant viruses which are as genetically stable, virulent and transmissible as the wild-type virus [30]. Here we report the results of a comprehensive study focusing on adamantane resistance in influenza A viruses circulating worldwide. Our results reveal a significant increase in adamantane-resistance frequencies in influenza A H1N1, H3N2, H5N1, H6, H7, and H9N2 viruses circulating during 2001–2013 (Table 7–12). The high levels of adamantane-resistance found in our study are comparable with other studies monitoring resistance worldwide [17, 18, 19, 31]. For example, adamantane resistance in A (H3N2) strains continued to increase in many countries from 2005 to 2007 (e.g. New Zealand, USA, Malaysia, and China) [31] (Table 8). However, resistance in A (H1N1) strains had been quite variable during that time period in some countries such as Japan, Australia, USA and Canada [15, 32, 33] (Table 7). The high frequency of adamantane-resistant variants indicates that continuous global surveillance and rapid identification of mutants are essential to monitor the emergence and spread of drug resistance, and to help with making informed decisions about antiviral usage in control of influenza virus infections.

To our knowledge, this study is the first attempt to evaluate the frequency and distribution of adamantane resistant influenza variants throughout the world over a period of 111 years. Results from our study provide further evidence that amino acid residues 26, 27, 30, 31, 34, and 38 in the M2 protein play a major role in determining the resistance phenotype of adamantanes of influenza A viruses and highlight the necessity of monitoring the susceptibility of influenza A viruses to antiviral drugs. Our findings raise concerns about the increasing prevalence of adamantane-resistant influenza variants and draw attention to the importance of tracking the emergence and worldwide spread of drug-resistant variants.

## Supporting Information

S1 Table Accession numbers and adamantane resistance mutations of M2 protein sequences from H1 subtype influenza A viruses used in this study. (XLSX)

S2 Table Accession numbers and adamantane resistance mutations of M2 protein sequences from H3 subtype influenza A viruses used in this study. (XLSX)

S3 Table Accession numbers and adamantane resistance mutations of M2 protein sequences from H5 subtype influenza A viruses used in this study. (XLSX)

S4 Table Accession numbers and adamantane resistance mutations of M2 protein sequences from H7 subtype influenza A viruses used in this study. (XLSX)

S5 Table Accession numbers and adamantane resistance mutations of M2 protein sequences from H9 subtype influenza A viruses used in this study. (XLSX)

S6 Table Accession numbers and adamantane resistance mutations of M2 protein sequences from H2, H4, H6, H10, H11, and H17 subtype influenza A viruses used in this study. (XLSX)
